# Effects and mechanisms of Tai Chi on mild cognitive impairment and early-stage dementia: a scoping review

**DOI:** 10.1186/s13643-023-02358-3

**Published:** 2023-10-28

**Authors:** Nibras Jasim, Darsiha Balakirishnan, Han Zhang, Genevieve Z. Steiner-Lim, Diana Karamacoska, Guo-Yan Yang

**Affiliations:** 1 School of Psychology, Western Sydney University, Penrith, NSW 2751 Australia; 2 School of Medical Science, Western Sydney University, Penrith, NSW 2751 Australia; 3 School of Acupuncture and Massage, Beijing University of Chinese Medicine, Beijing, 100029 China; 4 NICM Health Research Institute, Western Sydney University, Penrith, NSW 2751 Australia

**Keywords:** Tai Chi, Dementia, Mild cognitive impairment (MCI), Neurocognition, Systematic review

## Abstract

**Background:**

Dementia is associated with cognitive and functional decline that significantly impacts quality of life. There is currently no cure for dementia, thus, it is important to manage dementia in the early stages and delay deterioration. Previous studies have documented a range of health benefits of Tai Chi in people with early-stage dementia, however, none have systematically integrated these effects with their underlying mechanisms. The aims of this study were to (1) identify the neurocognitive, psychological, and physical health benefits of Tai Chi oi people with early-stage dementia, and (2) explore the underlying mechanisms of these effects.

**Methods:**

We searched systematic reviews (SRs) and randomised control trials (RCTs) on Tai Chi for adults aged 50 years and older with mild cognitive impairment (MCI) or early-stage dementia in MEDLINE, PubMed, Cochrane Library, EMBASE, and major Chinese databases. No language or publication restrictions were applied. Risk of bias was assessed.

**Results:**

Eight SRs with meta-analyses and 6 additional published RCTs revealed inconsistent findings of Tai Chi on improving global cognitive function, attention and executive function, memory and language, and perceptual-motor function. There was no significant between-group difference in depressive symptoms. The results from the RCTs showed that Tai Chi can reduce arthritis pain and slow the progress of dementia. No studies on MCI or early-stage dementia investigating the underlying mechanisms of Tai Chi were identified. Instead, nine mechanistic studies on healthy adults were included. These suggested that Tai Chi may improve memory and cognition via increased regional brain activity, large-scale network functional connectivity, and regional grey matter volume.

**Conclusion:**

The effects of Tai Chi on neurocognitive outcomes in people with MCI and early-stage dementia are still inconclusive. Further high-quality clinical trials and mechanistic studies are needed to understand if and how Tai Chi may be applied as a successful intervention to delay deterioration and improve the quality of life in people with an increased risk of cognitive decline.

**Supplementary Information:**

The online version contains supplementary material available at 10.1186/s13643-023-02358-3.

## Background

Dementia is a syndrome associated with over 100 different diseases where cognitive impairment interferes with physical and social functioning [1]. It is predicted that dementia will affect as many as 139 million people by 2050, compared to 55 million in 2020 [2]. The symptomatic prodromal phase of dementia, mild cognitive impairment (MCI), is characterised by a decline in cognitive function and relatively intact instrumental activities of daily living and is considered a transitional phase between neurotypical cognitive ageing and dementia [3, 4]. There are other health problems associated with MCI, such as increased falls risk [5], osteoarthritis and pain [6], poor balance [7], depression [8], and loneliness [9]. Approximately 35% of Australians aged 70 and older are estimated to have MCI, amongst which ~ 15% [10] will go on to develop dementia within 1–2 years, and up to 80% within 6 years [11]. MCI increases the risk of dementia > fivefold [12] and represents a stage for early intervention.

Currently, the benefits of pharmacological interventions are limited to symptomatic relief for people with dementia, with no approved pharmacological therapies for MCI [2, 13]. Therefore, non-pharmacological interventions have gained much attention for cognitive rehabilitation in MCI and dementia. Tai Chi is a traditional mind–body exercise originating in China in the seventeenth century A.D. that incorporates physical, cognitive, social, and meditative components in the same intervention [14, 15]. Traditionally, there are five major Tai Chi styles (i.e. *Chen*, *Yang*, *Wu*, *Wu/Hao*, and *Sun* styles), and with the development and broader use of Tai Chi, numerous newer styles, hybrids, and extensions become available to suit different needs and contexts [14, 15]. The intensity of Tai Chi practice is low to moderate with a set of flowing movements that suit the capacity of adults and older adults to practice for health and wellbeing [15].

Existing clinical studies investigating the benefits of Tai Chi for people with MCI and early-stage dementia have reported inconsistent findings. A systematic review found that Tai Chi is one of the mind–body interventions that can improve cognitive function (including memory) and activities of daily living, and results in a moderate reduction in falls risk, depression, stress, and dementia risk in people with MCI [14]. Similar findings on global cognitive function, memory, learning, and visuospatial perception enhancements in people with MCI were reported in another systematic review and meta-analysis [4]. However, another two recent meta-analyses [2, 16] found that Tai Chi was not superior to the control group in improving depressive symptoms and executive function in this population. Furthermore, it remains unclear which physical, psychological, and neurocognitive outcomes have and have not been investigated or well-evidenced in the existing literature on Tai Chi for early-stage dementia and MCI. Additionally, there is a gap in the literature relating to the underlying mechanisms of Tai Chi that may benefit older people with, or at risk of cognitive decline.

The potential mechanisms of Tai Chi for MCI and early-stage dementia have not been comprehensively summarised. Currently, several studies have identified the possible mechanisms of action of Tai Chi in other populations. For example, Tao et al. [1] reported that after practicing Tai Chi for 5 days per week for 12 weeks with each session lasting 60 min, the resting-state functional connectivity between the bilateral hippocampus and medial prefrontal cortex (mPFC) was significantly increased for healthy adults aged 50 to 70 years old compared with the health education control group. Uncovering the mechanisms of how Tai Chi works for people with MCI and early-stage dementia may help to clarify the relationship between intervention and diverse outcomes, aid with tailoring and refining interventions, optimise therapeutic effectiveness, and facilitate research translation to clinical practice [17].

This scoping review aimed to map the neurocognitive, psychological, and physical outcomes assessed in systematic reviews and randomised controlled trials on Tai Chi for people with MCI and early-stage dementia. In addition, we aimed to assess the effects and safety of Tai Chi on neurocognitive, physical, and psychological outcomes in these populations, and explore the underlying neuronal mechanisms.

## Methods

### Inclusion/exclusion criteria

#### Type of participants

Adults aged 50 years and older diagnosed with MCI or early-stage dementia, defined as mild Alzheimer’s disease or mild dementia, were included. No limitation on gender, ethnicity, or duration of cognitive decline was applied.

#### Type of interventions

All styles and forms of Tai Chi and training regimens were eligible, including traditional, modified, or simplified Tai Chi, Tai Chi pushing hands, and Tai Chi practiced with instruments (i.e. Tai Chi sword, Tai Chi knife, Tai Chi soft ball, and other forms). Single movement of Tai Chi, Tai Chi gait, and wheelchair/seated Tai Chi were also included. Limits on duration and frequency were not applied. Interventions combining Tai Chi with other practices with Tai Chi as the main component (e.g. 50 min of Tai Chi with 10 min of Qigong, meditation, or other form of exercise) were also included.

#### Type of controls

No intervention, wait-list control, usual care, and active control were all eligible for inclusion. Co-interventions were also included if all the study arms received the same co-intervention.

#### Type of evidence sources

To analyse the efficacy and safety of Tai Chi, we included systematic reviews (SRs) with meta-analyses of randomised controlled trials (RCTs) and/or non-randomised studies of interventions (NRSI). RCTs that met the criteria for participants, interventions, and controls, were also included if they were not identified in any included SRs and/or explored other outcomes which were not investigated by the included SRs.

To explore the potential mechanisms of Tai Chi, we included all relevant studies including systematic reviews, RCTs, and NRSIs.

### Information sources

We searched major English and Chinese databases from their inception to December 4, 2020, for potentially eligible articles, including MEDLINE, PubMed, Cochrane Library, EMBASE, China National Knowledge Infrastructure (CNKI), Chinese Scientific Journal Database (VIP), Sino-Med, and Wanfang Database. No language or publication restrictions were applied. A continual article search was conducted until January 10, 2022. No new articles were detected, and searching ceased to allow time for article finalisation.

The reference lists of all included articles were manually searched for additional eligible studies. Conference papers and dissertations were also searched electronically.

### Search strategy

Four reviewers (NJ, DB, HZ, and GYY) independently conducted the literature search, before working together in pairs. The search terms in English databases included (Tai Chi OR Taichi OR Tai ji OR Taiji OR Taijiquan OR Tai Chi Chuan) AND (Cognitive Impairment OR Mild Cognitive Impairment OR Cognitive Decline OR early-stage dementia OR mild dementia OR dementia risk factors OR memory OR brain function), as shown in Table 1.

**Table 1:** An example of the search strategy of PubMed

Item	Search terms
#1	(Tai chi[Title/Abstract] OR Taichi[Title/Abstract] OR Taiji[Title/Abstract] OR Tai ji[Title/Abstract] OR Tai chi chuan[Title/Abstract] OR Taijiquan[Title/Abstract])
#2	(Cognitive Impairment [Title/Abstract] OR Mild Cognitive Impairment [Title/Abstract] OR Cognitive Decline [Title/Abstract] OR early-stage dementia [Title/Abstract] OR mild dementia [Title/Abstract] OR dementia risk factors[Title/Abstract] OR memory[Title/Abstract] OR brain function[Title/Abstract])
#3	#1 AND #2

### Study selection

The reference manager software EndNote (version X9) was used to screen studies identified in English databases by two reviewers (NJ and DB) and NoteExpress (version 3.2) to screen studies from Chinese databases by another two reviewers (HZ and GYY). To maintain consistency, all reviewers performed calibration exercises according to the eligibility criteria before commencing the study selection process. After removing duplicates, the four reviewers worked in pairs and independently screened the titles/abstracts, followed by the full texts of all the articles against the eligibility criteria. The number and reasons for including and excluding studies were recorded and the screening results were compared. Any disagreements were resolved by discussion until a consensus was reached.

### Data extraction

A predefined form was used for data extraction. The extracted items included bibliometric information, participants' characteristics, details of Tai Chi and control group/interventions, and the main findings. For the mechanisms of Tai Chi, we extracted relevant quantitative and narrative data.

To improve consistency, all reviewers performed calibration exercises, as well as participated in the discussion of results and the data extraction manual prior to commencing the data extraction process. Four reviewers (NJ, DB, HZ, and GYY) independently extracted data using the pre-defined data extraction form. Any disagreements were resolved by discussion and achieving consensus.

### Quality assessment

The methodological quality of the included SRs was assessed with the critical appraisal tool A Measurement Tool to Assess systematic Reviews (AMSTAR 2) [18]. The Grading of Recommendations, Assessment, Development and Evaluation (GRADE) approach was used to grade the certainty of the systematic reviews and their reported measures of effect as ‘high’, ‘moderate’, ‘low’, or ‘critically low quality’ [19]. The risk of bias for the individual studies included in the SRs was evaluated according to the assessment provided in the SRs themselves.

The Cochrane risk of bias tool for randomised trials (RoB) was used to rate the methodological quality of included RCTs [20]. Here, RoB is structured into a fixed set of domains of bias, focussing on different aspects of trial design, conduct, and reporting. A proposed judgement about the risk of bias from each domain is determined based on answers to the signalling questions. Judgements can be ‘low’ or ‘high’ risk of bias or they can be rated as ‘unclear’ if the relevant information provided is not adequate to support the judgement.

To enhance consistency, all reviewers performed calibration exercises and discussed the results prior to appraising the quality of the included SRs and RCTs, as well as rating the certainty of the overall evidence. Four reviewers (NJ, DB, HZ, and GYY) collaborated in pairs and independently assessed the quality of the included studies. Any disagreements and discrepancies were resolved by discussion and reaching a consensus.

### Data synthesis and analysis

The Joanna Briggs Institute Manual for Evidence Synthesis: Chapter 11 Scoping Review [21] and the GRADE Handbook [19] were used to guide the data synthesis process of this scoping review. Frequency counts of populations, interventions, and characteristics of included studies are mapped in Table 2. The final assessment is reported in a summary of the key findings from the included SRs. No further analyses (i.e. meta-, network-, or re-analysis) were performed. The results are presented in a narrative format and in tables which include the important characteristics and the quality of the included studies. In addition, a summary of the estimates of effect for each main outcome, and the GRADE findings on the certainty of the evidence are also included.

**Table 2:** Characteristics of included systematic reviews

Author, year	Country	Disease/condition	Studies included (no. and study design)	Sample size^a^	Tai Chi intervention	Comparisons	Outcome and measurement
Farhang et al. (2019) [14]	Chile	Mild cognitive impairment	4 about Tai Chi (3 RCTs, 1 PNRCT)	529	2–3 × 30–90 min/week for 4–24 weeks	Stretching and relaxation exercise, no intervention, no TC practice, psychoeducation	1) Cognitive function: CDR, ADAS-cog 2) Executive function: DSF and DSB, TMT 3) Memory: The Logical Memory—delayed recall, HVLT, RBMT, RBMT-II, TEA 4) Visuospatial ability: Block Design Test
Zhang et al. (2020) [16]	China	Mild cognitive impairment	7 RCTs	1068	3 × 30–50 min/week for 12–52 weeks	Stretching, daily activity, health education	1) Global cognitive function: MMSE 2) Memory: Logical Memory Delayed Recall Test 3) Executive function: Digit Span Test (DST) Forward & Backward 4) Verbal fluency: CVFT 5) Visual span: Visual Span Test Forward and Backward or Block Design Test 6) Depressive symptoms: CSDD or GDS
Lim et al. (2019) [22]	Canada	Early-stage dementia	9 (6 RCTs, 2 NRCTs, 1 PNCT)	11–238	1–4 × 20–60 min/week for 8–52 weeks	Health talk group, handicraft, adapted physical group activity and education on health, no intervention, stretching and relaxation exercises, education group on cognitive impairment, health and cultural information class	1) Global cognition: MMSE, ADAS-Cog, CDR 2) Working memory and executive function: DSB, DSC, DSF, 15-Word immediate recall and/or TMT-B 3) Attention and concentration: Stroop Colour and Word, DSB 4) Verbal learning and memory: HVLT-R, 15-Word 30-Min Delayed Recall, RBMT 5) Self-perception of memory: MIC, SMC 6) Semantic memory: CVF 7) Visuospatial ability: Block Design Test
Wang et al. (2018) [23]	China	Cognitive impairment, mild cognitive impairment or dementia	7 about Tai Chi (2 RCTs, 2 CCS, 3 CCT)	808	2–3 × 30–90 min/week for 8–48 weeks	Health talk, handicraft, memory intervention program, muscle-stretching and toning exercise, usual lifestyle, usual care	1) Global cognition: MMSE, ADAS-cog, MoCA 2) Memory: MIC, immediate verbal recall, delayed verbal recall, HVLT, RBMT 3) Executive function: CVF, DSF, forward digit sequence, DSB, backward digit sequence, TMT-A and B
Wei et al. (2020) [2]	China	Mild cognitive impairment	12 (4 RCTs, 7 quasi-experimental studies, 1 nonrandomized control group pretest–posttest design)	981	2–5 × 30–60 min/week for 12–52 weeks	Maintain usual daily physical activities, related health education, stretching and toning exercise	1) Global cognitive ability: MMSE, MoCA, ADAS-Cog, CDR 2) Memory: AVLT-long-term delayed recall 3) Attention: DSB, DSF 4) Executive ability: TMT 5) Language domain of cognition: CVF 6) Visual-spatial cognition: block design test, visual span test 7) IADL: Lawton’s instrumental activities of daily living and functional activities questionnaire (FAQ) 8) Quality of life: SF-12, PD-39
Yang et al. (2020) [4]	China	Mild cognitive impairment	11 RCTs	1061	1–6 × 30–120 min/week for 10–48 weeks	Stretching and toning exercise, escitalopram plus health education, maintain routine daily activities, no intervention, nonathletic activities, educational information related to cognition, memory training	1) Global cognitive function: MMSE, MoCA, ADAS-Cog, MDRS, CDR-SOB 2) Memory and learning: delayed recall, DSF, DSB, California Verbal Learning Test, Rey Auditory Verbal Learning Test (immediate and delayed recall), Mattis memory score, logical memory-delayed recall score, and Wechsler Memory Scale 3) Mental speed and attention: visual span (forward), visual span (backward), Stroop Colour and Word Test, Mattis attention score, FAB, Chinese Trail A (seconds), Chinese Trail B (seconds), Trail Making Test A (errors), Stroop Colour and Word Test, Trails A Time (seconds), Trails B Time (seconds), TMT Part A, TMT Part B 4) Ideas, Abstraction, Figural Creations, and Mental Flexibility: CVF, Mattis conceptualization score, Mattis initiation score, category verbal fluency (animals), WAIS, and Trail-Making Test Part B-A score 5) Visuospatial perception: Rey Figure Test (recall), block design score, clock drawing test, bell cancellation test, Mattis construction score, and Rey Figure Test (copying)
Zheng et al. (2017) [3]	China	Mild cognitive impairment	3 RCTs	455	2–3 × 30–90 min/week for 20–48 weeks	Usual daily activities; stretching and relaxation exercises, memory intervention program	WAIS, RBMT, SMC, ADAS-cog, BBS, CSDD, CDR, CVF, DAD, DR, NPI, MMSE, VS, DTC, HVLT, RAPA, RBMT, SF-36, TEA, UG
Zou et al. (2019) [24]	China	Mild cognitive impairment	6 about Tai Chi (3 RCTs, 3 NRCTs)	789	2–4 × 30–90 min/week for 12–52 weeks	Stretching exercise, memory training, unaltered lifestyle, educational class	1) Global cognition: MMSE, CDR, MoCA 2) Executive function: TMT-B, DST-FB 3) Short-term memory: Delayed Recall Test, RBMT, RBMT-delayed 4) Cognitive processing speed: DST-F, DSST 5) Visuospatial ability: block design test

*Abbreviations: RCT* randomised controlled trial, *PNRCT* pilot non-randomised controlled trial, *NRCT* non-randomised controlled trial, *PNCT* prospective non controlled trial, *CCS* cluster controlled studies, *CCT* controlled clinical trials, *CDR* Clinical Dementia Rating, *CVFT* Category Verbal Fluency Test, *ADAS-cog* Alzheimer Disease Assessment Scale—Cognitive Subscale, *DSF* Digit Span forward, *DSB* Digit Span backward, *TMT* Trial-Making Test, *TMT-A* Trial-Making Test-Part A, *TMT-B* Trial-Making Test-Part B, *HVLT* Hopkins Verbal Learning Test, *HVLT-R* Hopkins Verbal Learning test–Revised, *RMBT* Rivermead Behavioural Memory Test, *TEA* Test of Everyday Attention, *MMSE* Mini-Mental Status Exam, *DSC* Digit Symbol Coding, *MIC* Memory Inventory for Chinese Questionnaire, *SMC* Subjective Memory Complaints Scale, *MoCA* Montreal Cognitive Assessment, *AVLT* Auditory Verbal Learning Test, *IADL* Instrumental Activities of Daily Living, *PD-39* Parkinson’s Disease Questionnaire-39, *MDRS* Mattis Dementia Rating Scale, *CDR-SOB* Clinical Dementia Rating Sum of Boxes, *FAB* Frontal Assessment Battery, *WAIS* Wechsler Adult Intelligence Scale, *BBS* Berg Balance Scale, *CSDD* Cornell Scale for Depression in Dementia, *CVF* category verbal fluency, *DAD*, Disability Assessment for Dementia, *DR* delayed recall, *NPI* Neuropsychiatric Inventory; *VS* visual span, *DTC* dual-task cost, *RAPA* Rapid Assessment of Physical Activity Scale, *SF-36* RAND 36-Item Short-Form Health Survey-Medical Outcomes Study, *UG* usual gait, *DST-FB* Digit Span Test Forward–Backward, *DSST* Digit Symbol Substitution Test, *GDS* Geriatric Depression Scale

^a^Sample size of the included Tai Chi studies

## Results

### Characteristics and quality of included studies

#### Characteristics

**Fig. 1:**
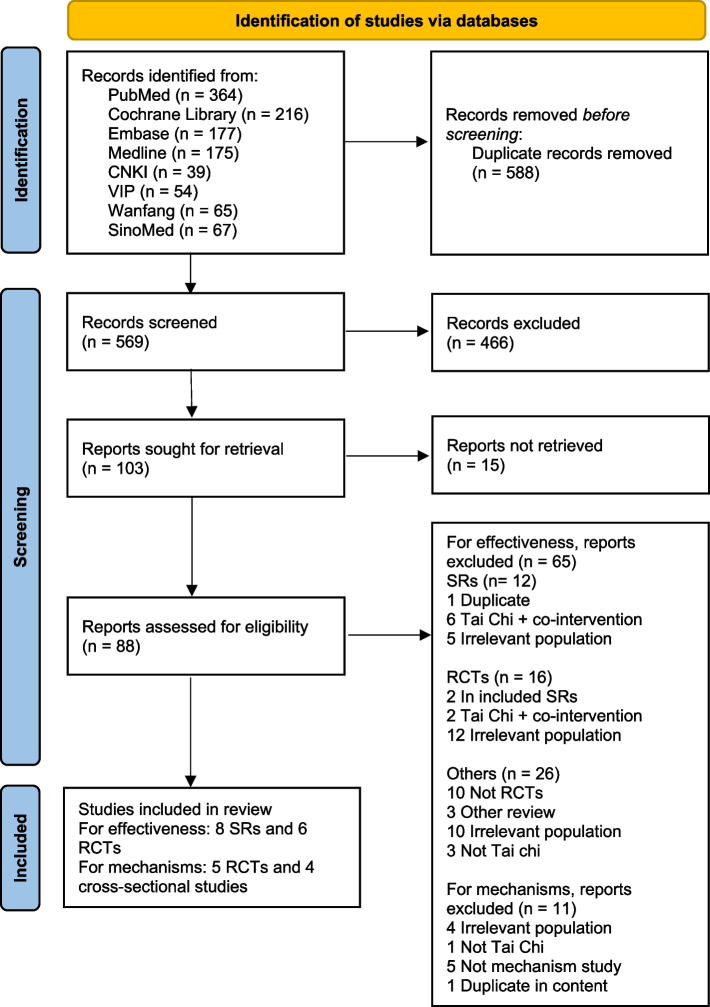
PRISMA 2020 flow diagram for new systematic reviews which included searches of databases and registers only

In total, 14 studies were included in this review, 8 SRs [2–4, 14, 16, 22–24] and 6 RCTs [25–30], as displayed in the flowchart in Fig. 1. A further 11 RCTs were identified within the included meta-analyses; however, these were excluded for the reasons demonstrated in Table S1 in Supplementary Materials.

The characteristics of the included SRs are summarised in Table 2. The 8 included SRs were published between 2017 and 2020, representing the most current evidence in this area. All 8 SRs searched English databases, of which 2 did not apply language limitation [3, 23] for their literature search. One SR also searched French databases [22] and 3 SRs also searched Chinese databases [2, 4, 24]. The majority of the searches were conducted from the inception of databases up to a few months preceding the publication of the SRs. The most recent search was conducted from inception to December 2019 [16].

Collectively, 5054 individuals were included in the 8 SRs, with the sample size of their included RCTs ranging from 11 to 1061. The diagnosis of the participants included were MCI (*n* = 7) or early-stage dementia (*n* = 1). Participants were aged between 55 and 85 years old. Tai Chi was included as part of an array of mind–body interventions (*n* = 4) or exclusively as an intervention (*n* = 4) for the experimental group. The control interventions included stretching and relaxation exercise (*n* = 7), health education (*n* = 5), usual lifestyle (*n* = 5), memory training (*n* = 4), no intervention (*n* = 3), or handicraft (*n* = 2). Each Tai Chi session ranged from 30 to 120 min for a weekly frequency that varied from 1 to 6 times. The duration of the intervention lasted 8 to 52 weeks. The outcomes that were most frequently reported across the 8 SRs were global cognition, memory, executive function, and perceptual-motor function.

The characteristics of the included 6 RCTs are summarised in Table 3. These studies were conducted in China (*n* = 1), USA (*n* = 2), Thailand (*n* = 2), and Turkey (*n* = 1). A total of 535 participants were included, with 223 in the intervention group and 312 in the control group. The sample size ranged from 42 to 261 individuals. All the included RCTs involved adults aged above 60 years old, with an average age ranging from 67.5 to 78.9 years. The participants were diagnosed with MCI, amnestic-MCI, or mild dementia. A range of diagnostic tools were used, including Mini-Mental State Examination (MMSE), Montreal Cognitive Assessment (MoCA), Clinical Dementia Rating (CDR), Diagnostic and Statistical Manual of Mental Disorders (DSM-IV), and Petersen’s criteria for MCI subtypes. All included RCTs had Tai Chi alone as the intervention, except one study in which Tai Chi was part of an integrated cognitive training and mind–body physical exercise plus nurse-led risk factor modification program. The sessions for the Tai Chi training varied from 20 to 40 min, which were practiced 2 to 3 times per week. The duration of the Tai Chi intervention ranged between 12 and 48 weeks. The prevalent outcomes measured across the included RCTs were cognitive function, pain, depression, balance, and falls risk.

**Table 3:** Characteristics of included randomised controlled trials

Author, year	Study design	Country	Disease/condition	Diagnostic criteria	Mean age (years) (T)	Mean age (years) (C)	Sample size total (T/C)	Frequency and duration of intervention (T)	Control group	Outcome/measurement
Okuyan and Deveci (2020) [26]	RCT	Turkey	Mild cognitive impairment	MMSE and MoCA of < 25 points	74.21 (6.93)	74.21 (6.93)	42 (20/22)	2 × 35–40 min/week for 12 weeks	Not subjected to any physical practice	1) Risk of falling: TAT (gait and balance) 2) Physical activity: PASE 3) Fear of movement: TSK 4) Behaviours related to falls: FaB scale
Lam et al. (2012) [25]	Single-blind cluster RCT	China	Risk of cognitive decline	CDR of 0.5 or Neuropsychological criteria for amnestic-mild cognitive impairment	77.2 (6.3)	78.3 (6.6)	261(92/169)	At least 3 × 30 min/week for 12 months	Muscle-stretching and toning exercises	1) Primary outcome: progression to dementia measured by DSM-IV criteria; cognitive test scores of Cantonese version of the ADAS-Cog, DS, delay recall, CVFT, TMT, MMSE 2) Secondary outcomes: CSDD assessed depressive symptoms in persons with cognitive impairment; NPI was used to assess changes in neuropsychiatric symptoms; BBS assessed functional balance
Sungkarat et al. (2017) [27]	Single-blind RCT	Thailand	Amnestic multiple-domain MCI (a-MCI)	Petersen’s criteria for diagnosing amnestic a-MCI, had a score of ≥ 24 on MMSE and < 26 on MCA	68.3 (6.7)	67.5 (7.3)	66 (33/33)	3 × 50 min/week for 15 weeks	Educational material covering information related to cognitive impairment and fall prevention	1) Primary outcome: Episodic memory was measured by LM-delayed recall; Visuospatial ability was assessed using the Block Design Test; Executive function was assessed using DSF, DSB, and TMT Part B–A 2) Secondary outcome: PPA composite fall-risk and component scores; edge contrast sensitivity was assessed using the Melbourne Edge Test; Proprioception was assessed using a lower limb matching test; Knee extension strength of the dominant leg was measured using a spring gauge; Simple hand reaction time was measured in milliseconds; postural sway was assessed using a sway meter that measured displacement of the body
Sungkarat et al. (2018) [28]	Assessor-blinded, prospective interventional RCT	Thailand	Amnestic multiple-domain MCI (a-MCI)	Petersen’s criteria for diagnosing amnestic a-MCI, had a score of ≥ 24 on MMSE and < 26 on MCA	68.3 (6.7)	67.5 (7.3)	56 (29/27)	3 × 50 min/week for 6 months	Educational material covering information related to cognitive impairment and fall prevention	1) Primary outcome: Memory was assessed using LM delayed recall; Visuospatial ability was assessed using the Block Design Test; executive function was assessed using DSF, DSB, and TMT B minus A (B-A) 2) Secondary outcome: plasma BDNF, TNF-α, and IL-10 levels
Tsai et al. (2013) [29]	Cluster-randomized clinical trial	USA	Moderate, mild, or subtle cognitive impairment	MMSE score of 18–28	78.89 (6.91)	78.93 (8.30)	55 (28/27)	3 × 20–40 min/week for 20 weeks	Health education, culture-related activities, and other social activities (e.g. sharing travel experiences, hobbies, and collections)	1) WOMAC was used to measure subjective pain, physical functioning, and stiffness 2) A modified GUG test was used to measure the elder’s speed in getting up from an armchair, walking as fast as he or she could for 50 feet, returning to the chair, and sitting down 3) STS test was modified for our elderly participants by asking participants, with arms across the chest, to rise five times from a chair as fast as possible 4) Cognitive functioning was measured by the MMSE
Tsai et al. (2015) [30]	Cluster-randomized clinical trial	USA	Moderate, mild, or subtle cognitive impairment	MMSE score of 18–28	78.89 (6.91)	78.93 (8.30)	55 (28/27)	3 × 20–40 min/week for 20 weeks	Attention control education group (instructor-led educational activities)	1) VDS for measuring pain in elders with cognitive impairment 2) Observation of pain behaviour: participants engaged in a series of daily tasks (sitting, standing, walking, and reclining), using Keefe’s observational method for OA knee pain 3) Analgesic intake: examined changes in analgesic intake

*Abbreviations*: *a-MCI* multiple-domain MCI, *RCT* randomised controlled trial, *TAT* Tinetti assessment tool, *PASE* physical activity scale for the elderly, *TSK* Tampa scale of kinesiophobia, *FaB* falls behavioral, *MMSE* Mini-Mental Status Exam, *BP* blood pressure, *PEF* peak expiratory flow, *COPD* chronic obstructive pulmonary disease, *CDR* Clinical Dementia Rating, *DAD* Disability Assessment for Dementia, *CSDD* Cornell Scale for Depression in Dementia, *NPI* Neuropsychiatric Inventory, *BBS* Berg Balance Scale, *MIC* Memory Inventory for Chinese Questionnaire, *ADAS-cog* Alzheimer Disease Assessment Scale—Cognitive Subscale, *CVFT* category verbal fluency test, *CMMSE* Cantonese version of mini-mental state examination, *NP* neuropsychiatric, *LM* Logical memory, *DS* Digit Span, *DSF* Digit Span forward, *DSB* Digit Span backward, *TMT* Trial-Making Test, *PPA* Physiological Profile Assessment, *BDNF* brain-derived neurotrophic factor, *TNF-α* tumor necrosis factor-α, *IL-10* interleukin-10, *WOMAC* Western Ontario and McMaster Universities Osteoarthritis Index, *GUG* get up and go, *STS* sit-to-stand, *GDS* Geriatric Depression Scale, *HK-MoCA* Montreal Cognitive Assessment Hong Kong version, *EQ-5D* EuroQoL 5-D Questionnaire, *EQ-VAS* EuroQoL visual analogue scale, *GDS-15* Geriatric Depression Scale with a maximum score of 15, *GAS-20* Geriatric Anxiety Scale with a maximum score of 20, *Mattis DRS* Mattis Dementia Rating Scale, *DQoL* Dementia Quality of Life, *VDS* Verbal descriptive scale

#### Quality assessment

##### AMSTAR 2

Four SRs with meta-analyses were included for data synthesis and their study quality was assessed by AMSTAR 2. As shown in Table 4, 3 of the 4 included SRs (75%) [4, 23, 24] were rated as critically low quality due to serious concerns with their protocol, meta-analysis, or study of RoB impacting their conclusions, as well as the assessment and discussion of publication bias. The other SR [2] was rated as low quality due to serious concerns in the assessment and discussion of publication bias. Regarding the protocol, only one SR [2] reported it was established prior to conducting their review as well as any deviations from the protocol, while the other 3 SRs did not report this information. Regarding the search strategy, only one SR [24] had a comprehensive literature search strategy by searching trial registries and reference lists of included studies, consulting content experts, as well as conducting the search within 24 months of completing the review. Regarding publication bias, 2 studies performed graphical tests and discussed its impact on the results of their review, while the other 2 studies did not report on publication bias.

**Table 4:** AMSTAR 2 quality rating of included systematic reviews

	PICO research question	Protocol	Study design inclusion rationale	Comprehensive literature search	Duplicate study selection	Duplicate data extraction	List excluded studies + rationale	Adequate study characteristics	Satisfactory RoB Assessment	Funding source of studies	Appropriate meta-analysis	Study RoB impact on meta-analysis	Study RoB impact on conclusions	Heterogeneity explained, discussed	Publication bias assessed, discussed	Conflict of interest, funding declared
Author, year	1	2	3	4	5	6	7	8	9	10	11	12	13	14	15	16
Wang et al. (2018) [23]	Y	N	N	PY	Y	Y	N	Y	Y	N	Y	N	N	Y	Y	Y
Wei et al. (2020) [2]	Y	Y	N	PY	N	N	N	Y	Y	N	Y	N	Y	Y	N	Y
Yang et al. (2020) [4]	Y	N	N	PY	Y	Y	N	PY	PY	N	N	N	N	N	N	Y
Zou et al. (2019) [24]	Y	N	Y	Y	Y	Y	N	Y	PY	N	N	N	N	Y	Y	N
Lim 2019	Y	N	N	PY	Y	Y	N	PY	PY	N	N	N	N	N	N	Y
Farhang 2019 [14]	Y	N	N	PY	Y	Y	Y	PY	PY	N	N	N	N	N	N	Y
Zheng 2017 [3]	Y	N	N	PY	Y	Y	N	PY	N	N	N	N	N	Y	N	N
Zhang et al. 2020 [16]	Y	N	N	PY	Y	Y	N	PY	Y	N	Y	N	N	Y	N	N

*Y* yes, *PY* partial yes, *N* no. *High quality* yes, for all critical and non-critical items. *Moderate quality* yes, or partial yes for all applicable critical items and yes or partial yes, for more than 4 applicable non-critical items. *Low quality* yes, or partial yes for more than 4 applicable critical items; and yes or partial yes, for more than 4 applicable non-critical items. *Critically low quality* yes or partial yes, for 4 or less applicable critical or non-critical items. *Critical items*: 2, 4, 9, 11, 13, and 15. *Non-critical items*: 1, 3, 5, 6, 7, 8, 12, 14, and 16

##### GRADE certainty

The GRADE certainty of effect estimates from the included SRs and meta-analyses is shown in Table 5. The evidence certainty for cognitive function and attention ranged from moderate to low, for memory ranged from high to very low, and for language and were moderate for perceptual-motor function and depressive symptoms.

**Table 5:** GRADE certainty of SRs and meta-analyses by main outcomes and measurements

Outcome	Estimate of effect [95% CI]	*I*^2^	No. of participants (studies)	Total score	Reason^1^	GRADE certainty
Global cognition						
Tai Chi vs (health talk, muscle-stretching and toning exercise, usual lifestyle, usual care, health education)	SMD 0.4 [− 0.13, 0.93]	88%	574 (5)	− 4	a, h, d	Very low ⨁◯◯◯
Tai Chi vs (stretching and toning exercise, related health education, usual daily activities)	MD 1.98 (1.32, 2.65)	74%	780 (8)	− 1	a	Moderate ⨁⨁⨁◯
Tai Chi vs (playing cards or singing, stretching and toning exercise, Escitalopram plus health education, education)	SMD 0.40 (0.08, 0.73)	79%	858 (5)	− 3	a, h	Very low ⨁◯◯◯
Tai Chi vs stretching and toning exercise	SMD 0.38 (0.22, 0.55)	0%	590 (2)	− 1	a	Moderate ⨁⨁⨁◯
Tai Chi vs (cognitive behaviour therapy, usual care, stretching, health education, recreational activities)	MD 0.29 (− 0.16, 0.74)	0%	785 (5)	− 1	d	Moderate ⨁⨁⨁◯
Tai Chi vs stretching and toning exercise	SMD 0.44 (0.24, 0.64)	27%	590 (2)	− 1	a	Moderate ⨁⨁⨁◯
Memory						
Tai Chi vs (health talk, muscle-stretching and toning exercise, health education)	SMD 0.40 (− 0.10, 0.90)	75%	379 (3)	− 3	a, b, d	Very low ⨁◯◯◯
Tai Chi vs (educational class, unaltered lifestyle, memory training)	SMD 0.77 (0.45, 1.09)	23.8%	226 (4)	− 2	f	Low ⨁⨁◯◯
Tai Chi vs (stretching, daily activity, health education)	MD 0.37 (0.13, 0.61)	7%	726 (4)	0		High ⨁⨁⨁⨁
Attention						
Tai Chi vs (maintain usual daily physical activities, stretching and toning exercise)	SMD 0.57 (− 0.25, 1.40)	74%	287 (2)	− 2	a, d	Low ⨁⨁◯◯
Tai Chi vs (maintain usual daily physical activities, stretching and toning exercise)	SMD 0.03 (− 0.22, 0.27)	0%	287 (2)	− 2	a, d	Low ⨁⨁◯◯
Executive function						
Tai Chi vs (muscle-stretching and toning exercise, Health education)	SMD 0.10 (− 0.16, 0.35)	13%	327 (2)	− 2	a, d	Low ⨁⨁◯◯
Tai Chi vs (maintain usual daily physical activities, stretching and toning exercise)	SMD 0.79 (− 1.08, 0.51)	0%	209 (3)	− 2	a, d	Low ⨁⨁◯◯
Tai Chi vs (stretching, daily activity, health education)	MD 0.03 (− 0.16, 0.22)	0%	726 (4)	− 1	d	Moderate ⨁⨁⨁◯
Verbal fluency						
Tai Chi vs stretching	MD 0.47 (− 0.76, 1.70)	0%	231 (2)	− 1	d	Moderate ⨁⨁⨁◯
Visual span						
Tai Chi vs (stretching, health education, daily activity)	SMD 0.57 (0.23, 0.91)	75%	726 (4)	− 1	b	Moderate ⨁⨁⨁◯
Depressive symptoms						
Tai Chi vs (stretching, cognitive behaviour therapy, usual care)	SMD 0 (− 0.14, 0.15)	0%	730 (4)	− 1	d	Moderate ⨁⨁⨁◯

^1^Reasons: risk of bias (*a* − 1: serious, *f* − 2: very serious); inconsistency (*b* serious, *h* very serious); indirectness (*c* serious, *i* very serious); imprecision (*d* serious, *j* very serious); publication bias (*e* serious, *k* very serious)

*a*, some concerns (one or two RoB categories > 75%); *b* heterogeneity *I*^2^ ≥ 75% or NI and all RCTs favour one direction, *d* MA sample size: ≥ 200 and 95%CI overlaps zero, *f* high risk of bias (all three RoB categories ≤ 75%), *h* heterogeneity *I*^2^ ≥ 75% or NI and mixed direction of results ± low overlap of CI (confirm with a visual inspection of Forest plot)

##### Risk of bias

The results of the risk of bias assessment for the 6 included RCTs are presented in Table 6. For random sequence generation, all 6 RCTs used appropriate random sequence generation methods. Four RCTs had applied allocation concealment [27–30], while 2 [25, 26] did not report this information. All the RCTs had reported blinding of the outcome assessment. None of the studies were found to selectively report the outcome. In terms of withdrawals and dropouts, 5 RCTs included the reason, while one RCT did not provide any information relating to this criterion.

**Table 6:** Risk of bias quality assessment of included randomised controlled trials

Author, year	Random sequence generation method	Allocation concealment	Blinding of outcome assessment	Selective outcome reporting	Withdraw/dropout
Lam 2012 [25]	Y	NI	Y	N	Y
Okuyan 2020 [26]	Y	NI	Y	N	Y
Sungkarat 2017 [27]	Y	Y	Y	N	Y
Sungkarat (2018) [28]	Y	Y	Y	N	Y
Tsai 2013 [29]	Y	Y	Y	N	Y
Tsai 2015 [30]	Y	Y	PY	N	NI

*Abbreviations*: *Y* yes, *PY* partial yes, *N* no, *NI* no information

### Effects of Tai Chi: evidence from meta-analyses

The five included SRs with meta-analyses [2, 4, 16, 23, 24] investigated the effects of Tai Chi on neurocognitive and psychological outcomes for people with MCI (Table 7).

**Table 7:** Mapping of outcomes and summary of main findings of included SRs and meta-analyses

Outcome	Study ID	Disease/condition	Instruments (no. of studies)	Intervention vs control (no. of participants)	Estimate of effect (95% CI), p, *I*-square	GRADE certainty
Global cognition	Wang et al. (2018) [23]	Cognitive impairment, mild cognitive impairment or dementia	MMSE; ADAS-cog; MoCA (5: 1 RCT, 4 CCT)	TC (*n* = 249) vs health talk, muscle-stretching, and toning exercise, usual lifestyle, usual care, health education (*n* = 325)	SMD = 0.40 (− 0.13, 0.93), 0.14, 88%	Very low
	Wei et al. (2020) [2]	Mild cognitive impairment	MMSE; MoCA; ADAS-Cog (8: 4 RCT, 4 non-RCT)	TC (*n* = 353) vs stretching and toning exercise, related health education, maintaining usual daily physical activities (*n* = 427)	MD = 1.98 (1.32, 2.65), 0.00001, 74%	Moderate
	Yang et al. (2020) [4]	Mild cognitive impairment	MMSE (5 RCTs)	TC (*n* = 363) vs stretching and toning exercise, Escitalopram plus health education, nonathletic activities (playing cards or singing), education (*n* = 495)	SMD = 0.40 (0.08, 0.73), 0.02, 79%	Very low
	Yang et al. (2020) [4]	Mild cognitive impairment	ADAS-Cog (2 RCTs)	TC (*n* = 363) vs stretching and toning exercise (*n* = 227)	SMD = 0.38, (0.22, 0.55), 0.00001, 0%	Moderate
	Zhang et al. (2020) [16]	Mild cognitive impairment	MMSE (5 RCTs)	TC (*n* = 325) vs cognitive behaviour therapy, usual care, stretching, health education, recreational activities (*n* = 460)	MD = 0.29, (− 0.16, 0.74), 0.21, 0%	Moderate
	Yang et al. (2020) [4]	Mild cognitive impairment	CDR-SOB (2 RCTs)	TC (*n* = 363) vs stretching and toning exercise (*n* = 227)	SMD = 0.44, (0.24, 0.64), 0.0001, 27%	Moderate
Memory	Wang et al. (2018) [23]	Cognitive impairment, mild cognitive impairment or dementia	MIC; delayed recall; Logical memory delayed recall (3: 2 RCT, 1 CCT)	TC (*n* = 152) vs Health talk (nonactive attention placebo), Muscle-stretching and toning exercise, health education (*n* = 227)	SMD = 0.40 (− 0.10, 0.90), 0.11, 75%	Very low
	Zou et al. (2019) [24]	Mild cognitive impairment	Short-term memory: Delayed Recall Test, the Rivermead Behavioral Memory Test (4: 2 RCT, 2 CCT)	TC (*n* = 226) vs educational class, unaltered lifestyle, memory training (not specified)	SMD = 0.77 (0.45–1.09), 0.001, 23.8%	Low
	Zhang et al. (2020) [16]	Mild cognitive impairment	Delayed Recall test (4 RCTs)	TC (*n* = 297) vs stretching, daily activity, health education (*n* = 429)	MD = 0.37 (0.13, 0.61), 0.002, 7%	High
Attention	Wei et al. (2020) [2]	Mild cognitive impairment	Digit span test (forward) (2: 1 RCT, 1 non-RCT)	TC (*n* = 105) vs maintaining usual daily physical activities, stretching, and toning exercise (*n* = 182)	SMD = 0.57 (− 0.25, 1.40), 0.17, 74%	Low
	Wei et al. (2020) [2]	Mild cognitive impairment	Digit span test (backward) (2: 1 RCT, 1 non-RCT)	TC (*n* = 105) vs maintaining usual daily physical activities, stretching, and toning exercise (*n* = 182)	SMD = 0.03 (− 0.22, 0.27), 0.83, 0%	Low
Executive function	Wang et al. (2018) [23]	Cognitive impairment, mild cognitive impairment or dementia	Digit span forward–backward; TMT B minus A (B-A), digit span; CVFT; Chinese TMT-A; Chinese TMT-B (2: 1 RCT, 1 CCT)	TC (*n* = 125) vs muscle-stretching and toning exercise, health education (*n* = 202)	SMD = 0.10 (− 0.16, 0.35), 0.46, 13%	Low
	Wei et al. (2020) [2]	Mild cognitive impairment	The trail-making test (3: 1 RCT, 2 non-RCT)	TC (*n* = 102) vs maintaining usual daily physical activities, related health education (*n* = 107)	SMD = − 0.79 (− 1.08, − 0.51), 0.00001, 0%	Low
Performance ability	Zhang et al. (2020) [16]	Mild cognitive impairment	Digit Span Test (DST) (4 RCTs)	TC (*n* = 297) vs stretching, daily activity, health education (*n* = 429)	MD = 0.03 (− 0.16, 0.22), 0.77, 0%	Moderate
Verbal fluency	Zhang et al. (2020) [16]	Mild cognitive impairment	Category Verbal Fluency Test (CVFT) (2 RCTs)	TC (*n* = 231) vs stretching (*n* = 363)	MD = 0.47 (− 0.76, 1.70), 0.45, 0%	Moderate
Visual span	Zhang et al. (2020) [16]	Mild cognitive impairment	Visual Span Test (VST) or Block Design Test (BDT) (4 RCTs)	TC (*n* = 297) vs stretching, health education, daily activity (*n* = 429)	SMD = 0.57 (0.23, 0.91), 0.0009, 75%	Moderate
Depressive symptoms	Zhang et al. (2020) [16]	Mild cognitive impairment	Geriatric Depression Scale (GDS) or Cornell Depression Score (CDS) (4 RCTs)	TC (*n* = 297) vs stretching, cognitive behaviour therapy, usual care (*n* = 433)	SMD = 0 (− 0.14, 0.15), 0.95, 0%	Moderate

*Abbreviations*: *ADAS-cog* Alzheimer's Disease Assessment Scale-cognitive, *CVFT* Category Verbal Fluency Test, *CDR-SOB* Clinical Dementia Rating Scale Sum of Boxes, *MIC* Mini-Cog, *MMSE* Mini-Mental State Examination, *MoCA* Montreal Cognitive Assessment, *TMT* Trail Making Test

#### Neurocognitive outcomes

##### Global cognitive function

Global cognitive function was measured by Mini-Mental State Examination (MMSE), Montreal Cognitive Assessment (MoCA), and Alzheimer’s Disease Assessment Scale-Cognitive Subscale (ADAS-Cog) to investigate the effects of Tai Chi intervention compared to the control groups in four meta-analyses [2, 4, 16, 23]. The control groups include stretching and toning exercise, relevant health education, maintaining usual daily physical activities, playing cards, or singing, and Escitalopram plus health education. Tai Chi improved global cognition compared to control groups in two meta-analyses [2, 4], but no statistically significant differences between the groups were found in the other two meta-analyses [16, 23].

##### Attention and executive function

One meta-analysis [2] measuring attention and working memory used digit span forward and digit span backwards. The meta-analysis identified improved attention in the Tai Chi and control groups; however, this was not statistically significant. Furthermore, attention and executive function that was measured by Digit Span (forward and backward) [16, 23], processing speed and cognitive control by the Chinese Trail-Making Test (TMT) A and B [23], and verbal fluency by Category Verbal Fluency Test (CVFT) [23] were improved in the intervention groups compared to the control groups; again the improvement was not statistically significant. In contrast, processing speed and cognitive control (TMT) in the other meta-analysis [2] was statistically significant, favouring the Tai Chi intervention.

##### Memory

In addition, short-term memory that was measured by Logical Memory Delayed Recall Test [16, 24] and Rivermead Behavioural Memory Test [24] reported statistically significant improvement in the intervention groups. However, when it was measured by Logical Memory Delayed Recall Test, the between-group differences of the changes in memory were not statistically significant [23].

##### Language and perceptual-motor function

Moreover, one meta-analysis [16] reported that Tai Chi improved verbal fluency that was measured by Category Verbal Fluency Test (CVFT) and executive function that was measured by Digit Span (forwards and backwards), but the between-group differences were not statistically significant. In contrast, Tai Chi was superior to the control group in improving the Visual Span Test (visuospatial ability and visual attention) that was measured by Visual Span Test or Block Design Test [16].

#### Psychological outcomes

One meta-analysis reported beneficial effects of Tai Chi for depressive symptoms that were measured by the Geriatric Depression Scale (GDS) or Cornell Scale for Depression in Dementia (CSDD) in people with MCI. However, the between-group differences were not statistically significant [16].

### Effects of Tai Chi: evidence from RCTs

Six RCTs evaluating the effects of Tai Chi on people with MCI, which were not included in any of the included SRs, were identified and analysed in this review (Table 8).

**Table 8:** Clinical evidence summary for main outcomes of RCTs

Study ID	Intervention vs control (no. of participants)	Outcome/instrument(s)	*P* value
Lam et al. (2012) [25]	TC (*n* = 92) vs muscle-stretching and toning exercises (*n* = 169)	1) Progression to dementia: DSM-IV criteria	.06/.04*
		2) Depressive symptoms: The Cornell Scale for Depression (CSDD)	.17/.02*
		3) Changes in neuropsychiatric symptoms: The Chinese Neuropsychiatric Inventory (NPI)	.41/.14*
		4) Balance: The Berg Balance Scale (BBS)	.05/.02*
Okuyan and Deveci (2020) [26]	TC (*n* = 20) vs not subjected to any physical practice (*n* = 22)	1) Risk of falling in people with MCI: TAT includes:	
		- Tinetti balance assessment	0.000
		- Tinetti gait assessment	0.000
		2) Status of physical activity in people with MCI: PASE	0.000
		3) Fear of movement: TSK with 17 items	0.000
		4) Behaviours related to falls in people with MCI: The FaB scale	0.000
Sungkarat et al. (2017) [27]	TC (*n* = 33) vs educational material covering information related to cognitive impairment and fall prevention (*n* = 33)	1) Executive function was assessed using:	
		- Digit Span forward	0.43
		- Digit Span backward	0.43
		- Block design score	0.01
		2) Composite fall-risk and component scores: Physiological Profile Assessment (PPA) comprises a series of 5 sensorimotor assessments:	0.015
		- Edge contrast sensitivity	0.21
		- Lower limb proprioception	0.002
		- Knee extension strength	0.008
		- Hand reaction time	0.04
		- Postural sway	0.009
Sungkarat et al. (2018) [28]	TC (*n* = 29) vs educational material covering information related to cognitive impairment and fall prevention (*n* = 27)	1) Memory: Logical Memory (LM) delayed recall	0.01
		2) Visuospatial ability: Block Design Test	0.06
		3) Secondary outcomes:	
		- Plasma BDNF (Brain-derived neurotrophic factor)	0.04
		- TNF-α (tumor necrosis factor-α)	0.50
		- IL-10 levels (interleukin-10)	0.29
Tsai et al. (2013) [29]	TC (*n* = 28) vs health education, culture-related activities, and other social activities (e.g. sharing travel experiences, hobbies, and collections) (*n* = 27)	1) WOMAC was used to measure:	
		- subjective pain	0.006
		- physical functioning	0.021
		- stiffness	0.010
		2) A modified Get Up and Go (GUG) test	0.126
		3) Sit-to-Stand (STS) test	0.728
Tsai et al. (2015) [30]	TC (*n* = 28) vs attention control education group (instructor-led educational activities) (*n* = 27)	1) The verbal descriptive scale (VDS)	0.032
		2) Observation of pain behaviour	0.522
		3) Analgesic intake	0.062

*Abbreviations*: *ADAS-cog* Alzheimer’s Disease Assessment Scale-cognitive, *CDR-SOB* Clinical Dementia Rating Scale Sum of Boxes, *FaB* The Falls Behavioural scale, *PASE* Population Physical Activity Scale for the Elderly, *TSK* The Tampa Scale for Kinesiophobia, *WOMAC* The Western Ontario and McMaster Universities Arthritis Index

^*^Group difference at 1 year (*p* values, intention to treat analysis/per protocol analysis)

#### Neurocognitive outcomes

##### Global cognitive function

Tai Chi significantly improved visuo-spatial reasoning (block design) (*p* = 0.01) but was not superior in improving digit span (forward or backward) (*p* = 0.43) [27]. In addition, Tai Chi improved memory (*p* = 0.01), as measured by Logical Memory Delayed Recall [28]. Although the Tai Chi group had higher block design scores than the control group, this difference was not significant (*p* = 0.06) [28].

#### Physical outcomes

##### Pain

Components of the Western Ontario and McMaster Universities Arthritis Index (WOMAC) were significantly enhanced after the Tai Chi intervention compared to the control group [29]. The WOMAC components of subjective pain, physical functioning, and stiffness gradually improved over the 21-week intervention (*p* = 0.01, 0.02, and 0.01, respectively) [29]. The pain measured by the Verbal Descriptive Scale (VDS) in people with moderate, mild, or subtle cognitive impairment was significantly reduced in the Tai Chi compared to the control group (*p* = 0.03) [30]. However, the Tai Chi group’s observation of pain behaviour, measured by an observant assessor, and their analgesic intake did not significantly differ from the control group [30].

##### Balance

Tai Chi significantly improved balance for people with MCI (measured by the Berg Balance Scale; BBS), compared to the control group (*p* = 0.02), for both intention-to-treat and per-protocol analyses [25]. In addition, Tai Chi intervention significantly reduced the risk of falling and fear of movement while improving the Status of Physical Activity and Falls Behavioural Scale (FaB) scores in people with MCI with *p* < 0.01 [26]. Moreover, Tai Chi can potentially reduce falls for people with MCI, as assessed with the Physiological Profile Assessment (PPA) indicating the proprioception, muscle strength, reaction time, and postural sway, the overall PPA scores were significantly improved after the intervention (*p* = 0.02) [27]. The Get Up and Go (GUG) test used to measure participants’ speed of getting up from an armchair, walking as fast as possible for 50 feet, and then returning to the chair and sitting down, did not significantly change after treatment (*p* = 0.13) [29]. Sit-to-Stand (STS) test was modified for participants by asking them to rise 5 times from a chair as fast as possible with arms across the chest, which did not yield a significant difference at the end of the intervention [29].

#### Blood tests

Plasma brain-derived neurotrophic factor (BDNF) level was significantly increased for the Tai Chi group compared to that of the control (*p* = 0.04); whereas the plasma levels of the pro-inflammatory cytokine, tumor necrosis factor-α (TNF-α), and anti-inflammatory cytokine, interleukin-10 (IL-10), did not significantly differ between the 2 groups, *p* = 0.50 and 0.29, respectively [28].

#### Psychological outcomes

Cornell Scale for Depression in Dementia (CSDD) scores lowered by 49% for the intervention group (*p* = 0.02) per-protocol analysis, which statistically signifies an improvement in depressive symptoms [25].

#### Progression of dementia

After 1 year of practicing Tai Chi for at least 30 min per session and at least three sessions per week, Tai Chi was found to be superior to the control group (stretching and toning exercise) in slowing the progress of dementia as characterised by the DSM IV in people with amnestic MCI (*p* = 0.04) [25]. The authors reported that there were no changes in Neuropsychiatric Inventory (NPI) scores across time [25].

### Mechanisms

Our search (Table 1) did not produce any studies that investigated the mechanisms underlying the effects of Tai Chi in MCI or early-stage dementia. However, within the search terms, nine studies investigating the mechanisms in healthy adults were found. The potential mechanisms that underlie the effects of Tai Chi on neurocognitive, physical, and psychological outcomes were explored in five RCTs, one quasi-experiment, and three cross-sectional studies, as presented in Table S2 in Supporting Materials. We report the outcomes here in the interest of extending knowledge on how Tai Chi might confer neurocognitive, psychological, and physical benefits, that may be of use for the design and implementation of future Tai Chi dementia research.

Three broad categories of imaging protocols were used in the studies to identify Tai Chi-related brain changes: brain activity, functional connectivity, and structural changes. It should be noted that the participants in the nine studies were healthy adults without MCI or dementia. All nine studies investigated older participants, except for one RCT involving college students [31] and one cross-sectional study involving long-term Tai Chi practitioners aged 18 to 35 years old [32].

#### Brain activity

Two studies investigated whether Tai Chi modulated changes to the fractional amplitude of low-frequency fluctuations (fALFF) using functional magnetic resonance imaging (fMRI) to prevent age-related memory decline. One found, compared to the control group that received basic health education, that 12 weeks of Tai Chi increased fALFF in the dorsolateral prefrontal cortex (DLPFC) of participants in both the typical low frequency (0.010–0.080 Hz) and slow-5 (0.010–0.027 Hz) ranges [33]. Improved memory was associated with greater low-frequency and slow-5 fALFF changes in the medial PFC (mPFC), and the DLPFC (for low-frequency fALFF only). The second study revealed that there was a significant decrease in the fALFF values in the bilateral frontoparietal network, default mode network (DMN), and the anterior cingulate-dorsal prefrontal-angular gyri network of Tai Chi practitioners compared to controls [34]. Further, larger fALFF values in the frontoparietal network were linked with greater cognitive control (measured by reaction time in the attention network task) in Tai Chi practitioners and the intensity of Tai Chi practice was associated with higher fALFF values in the DMN. Another fMRI study showed that older women with 6 years of Tai Chi experience (vs. 6 years walking control group) had greater spontaneous regional homogeneity activation in temporal regions including the fusiform gyrus and hippocampus [35]. A functional near-infrared spectroscopy (fNIRS) study found increased activity (wavelet amplitudes) in both resting and movement states in the PFC, motor cortex, and occipital cortex in long-term Tai Chi practitioners compared to controls [36].

#### Functional connectivity

Six studies explored the effects of Tai Chi on functional connectivity to test potential mechanisms underpinning changes in cognition. Xie et al.’s [36] fNIRS study found increased global functional connectivity (phase coherence, coupling strength, and direction) in both resting and movement states in the Tai Chi compared to the control group. Another study showed that Tai Chi was associated with a significant increase in resting state functional connectivity between the posterior cingulate cortex (PCC) and the right putamen/caudate, in comparison to the control group [37]. Tai Chi-related improvements in overall memory (Weschler Memory Scale memory quotient; WMS MQ) and visual memory were associated with increased connectivity in the right temporal pole and mPFC [37]. Another showed that 12 weeks of Tai Chi Chuan improved resting state functional connectivity between the bilateral hippocampus and mPFC compared to the control group; this was positively associated with improved memory function (WMS-MQ) across all participants [1]. Compared with general aerobic exercise, 8 weeks Tai Chi practice enhanced resting state functional connectivity between the left middle frontal gyrus and the left superior parietal lobule [31]. Conversely, [38] fMRI study found that, compared to the control, the Tai Chi group demonstrated a significant *decrease* in resting state functional connectivity between the DLPFC and the left superior frontal gyrus (SFG) and anterior cingulate cortex (ACC) after 12 weeks training. Additionally, mental control scores were negatively associated with functional connectivity between the DLPFC and the left putamen. Another study showed that there was no significant difference between long-term Tai Chi practitioners and a Tai Chi-naïve control group in resting state DMN functional connectivity [32].

#### Structural changes

Three MRI studies reported the effects of Tai Chi exercise on brain plasticity by measuring changes in grey and white matter volume, and white matter tracts. The first study found that compared to the control group and general aerobic exercise, 8 weeks of Tai Chi training significantly increased the grey matter volume of the left middle occipital gyrus, left precuneus, left superior temporal gyrus, and the right middle temporal gyrus in college students [31]. One study above also reported that healthy older women who had practiced Tai Chi for over 6 years had higher grey matter density in inferior and medial temporal regions, including the hippocampus, compared to the walking control group [35]. Another study observed no significant difference in white matter tract integrity (measured by fractional anisotropy using MRI diffusion-weighted imaging) between Tai Chi and control groups [32].

## Discussion

### Summary of evidence

To the best of our knowledge, this scoping review is the first to comprehensively evaluate SRs, meta-analyses, and RCTs on the effects of Tai Chi on neurocognitive, physical, and psychological outcomes in individuals with MCI and early-stage dementia and explore its underlying mechanisms. The health outcomes investigated in the included SRs and RCTs were mainly neurocognitive outcomes, including global cognition function, attention and executive function, memory and language, and perceptual-motor function. Several psychological and physical outcomes were also assessed. The findings from the meta-analyses suggested that Tai Chi has positive effects on global cognition (moderate to very low certainty), memory (high to very low certainty), attention and executive function (moderate to low certainty), language and perceptual-motor function (moderate certainty), and depressive symptoms (moderate certainty) amongst people with MCI and early-stage dementia; the meta-analyses did not assess the physical outcomes.

The meta-analysis showed that Tai Chi had a favourable effect on improving global cognition and various cognitive domains. For example, Tai Chi was superior to muscle stretching and toning exercises in improving global cognition [2, 4, 16] and superior to educational classes in improving memory and executive functions in people with MCI. A potential explanation of Tai Chi’s effects is that mind–body exercise outperforms conventional physical exercise and health education in regulating mood and depression which are crucial risk factors for cognitive decline in people with MCI [23]. Discrepancies in the results might be caused by the variations of control groups, targeted populations, intervention designs of the included studies, and measurements used to evaluate these outcomes. For example, one meta-analysis included studies that utilised playing cards, singing, stretching and toning exercise, Escitalopram plus health education or education as their control groups [4]; whereas another utilised health education muscle stretching and toning exercise, usual lifestyle, and usual care [23]. Another possible reason for the discrepancy could be due to the type of the included studies. For example, 2 meta-analyses included only RCTs [4, 16], while other meta-analyses included both RCTs and non-RCTs [2, 23, 24]. One more possible reason for the results’ discrepancy is the targeted populations, which varied from MCI [2, 24] to early-stage dementia [4], and a combination of cognitive impairment, MCI, or dementia [23]. The variety of the measurements utilised in the meta-analyses can be another reason for the inconsistent results. There were various global cognition measures were used across studies (MMSE, MoCA, ADAS-Cog); however, not all these measures have good sensitivity and specificity in the populations of interest. For instance, the MoCA has the strongest evidence to discriminate people with MCI from cognitively normal older people and those with dementia, yet it was only adopted by 2 studies [2, 23].

From individual RCTs, Tai Chi was demonstrated to be beneficial in slowing the progress of dementia and improving depressive symptoms in people with MCI [25]. However, the effects of Tai Chi on global cognition function outcomes [27, 28, 30], and physical outcomes, including pain [29, 30], balance [25–27, 29], and blood test outcomes [28] yielded inconsistent results. This discrepancy could be due to the differences in outcome measurements, duration of practice in each session, or variety of controls (Table 3).

Tai Chi was found to consistently increase frontal activity, fronto-temporal functional connectivity, and hippocampal volume across most studies. Improvements in memory and cognitive control associated with Tai Chi were driven by increased activity in the mPFC, DLPFC, and fronto-parietal network (which encompasses the PFC) [34, 36, 38, 39]. Tai Chi-related memory enhancements were also related to increased mPFC and temporal/hippocampal functional connectivity [1, 37]. Further, Tai Chi practice was linked to greater grey matter volume across occipital, parietal, and temporal regions including the hippocampus [31, 35], and enhanced fusiform gyrus and hippocampal activation [35]. Together, findings suggest that Tai Chi enhances frontal cognitive control mechanisms, most likely due to focused attention on motor sequence learning and introspection (meditation, breathing) [40, 41] and this may strengthen learning and memory processes, reflected in neuroplastic changes in fronto-temporal connectivity and hippocampal volume. Deterioration in frontal executive functions is strongly linked with loss of instrumental activities of daily living [42], suggesting that Tai Chi may confer benefits that support older people to maintain independence with everyday activities through frontal cortical changes (e.g. larger DLPFC volumes as demonstrated for physical activity in older people [43]). Tai Chi’s capacity to upregulate functional brain plasticity in fronto-hippocampal networks may be underpinned by increases in neurogenic mechanisms such as brain-derived neurotrophic factor (BDNF) [44], which should be measured in future research along with hippocampal subfield analysis to determine the role of the dentate gyrus (a key regulator of neurogenesis).

### Limitations of this study

There are some limitations in the present review that should be acknowledged. First, although there was no language limitation of included studies, the search was only conducted from major English and Chinese databases so there is a potential language bias involved with the included studies in this review. Second, as our summary of findings is based on the effect estimates extracted from included SRs with meta-analyses, it limits our ability to appraise the quality of RCTs or non-RCTs pooled in the meta-analyses. This was mitigated by conducting AMSTAR ratings for SRs and GRADE certainty for these effect estimates, which can minimise the bias when interpreting the results.

### Methodological challenges and implications for future research

There are several methodological challenges identified in this review and recommendations for future research to draw stronger conclusions about the effectiveness of Tai Chi on the physical, mental, and neurocognitive outcomes of people with MCI and early-stage dementia. It is worth noting that the meta-analyses about Tai Chi for cognition were not exclusively studies on people with MCI. The combination of people with cognitive impairment, healthy adults, and early-stage dementia increased the clinical and statistical heterogeneity. It is recommended for future systematic reviews and meta-analyses to investigate the effect of Tai Chi exclusively on people with MCI or early-stage dementia. In addition, some of the meta-analyses included non-RCTs and quasi-experiments in their analyses. Considering more RCTs on Tai Chi for MCI are available, it is recommended for future researchers to analyse high-quality RCTs to increase the certainty of their conclusions. There was also only one meta-analysis investigating the effect of Tai Chi on depressive symptoms and one RCT investigating the effect on anxiety and depressive symptoms for people with MCI. It is recommended for future studies to conduct more RCTs and meta-analyses to investigate this effect for people with MCI and early-stage dementia to draw stronger conclusions. The available literature investigating the mechanisms of Tai Chi and its benefits on neurocognitive changes assessed by MRI and fMRI has only included indirect populations, such as healthy adults and college students, but not people with MCI or early-stage dementia. Future research should conduct RCTs involving direct populations using MRI and fMRI procedures to investigate the effect and mechanism of Tai Chi, particularly for people with MCI and early-stage dementia. Finally, the general methodological quality of included RCTs and those included in the SRs and meta-analyses was low, due to unclear bias of randomisation, which decreased the certainty of the evidence. Future studies should follow the reporting guideline CONSORT statement [39] to report RCTs, especially the methods of randomisation.

### Implications for clinical practice

This present review identified the positive effects of Tai Chi for a set of neurocognitive outcomes including cognition and memory, as well as several physical and mental health outcomes in people with MCI. Collectively, we found that an intervention period of at least 12 weeks with a frequency of 2 to 3 sessions a week, each lasting 30 to 60 min, was the most common duration reported in the included studies. However, no specific Tai Chi program can be recommended until more longer-term, higher-quality studies for the target population are available. It is noteworthy that, due to the poor methodological quality, small sample size, and inconsistent findings among included studies, we could not make a conclusive recommendation about the effects of Tai Chi on the management of cognitive and memory decline in people with MCI and early-stage dementia.

## Conclusion

Tai Chi seems to be beneficial in improving a set of neurocognitive outcomes, including global cognitive function, memory and attention, and several physical and psychological outcomes in adults with MCI. However, the findings are inconclusive because of poor quality of evidence and inconsistent findings. The mechanisms of how Tai Chi works remain unclear due to indirect evidence. More well-designed, large-scale, and transparently reported RCTs and meta-analyses for people with MCI or early-stage dementia are needed to inform clinical decision-making.

### Supplementary Information

**Additional file 1: Tablet S1.** Excluded studies that were screened for effectiveness analysis and reasons of exclusion. **Table S2.** Characteristics of included studies on the mechanisms of Tai Chi.

## Data Availability

The datasets supporting the conclusions of this article are included within the article and its additional files.

## Abbreviations

ACC: Anterior cingulate cortex
ADAS-cog: Alzheimer’s Disease Assessment Scale-cognitive subscale
AE: Aerobic exercise
AVLT: Auditory Verbal Learning Test
AMSTAR 2: A Measurement Tool to Assess systematic Reviews
BBS: Berg Balance Scale
BDNF: Brain-derived neurotrophic factor
BP: Blood pressure
C: Control
CCS: Cluster controlled studies
CCT: Controlled clinical trials
CDR: Clinical Dementia Rating
CDR-SOB: Clinical Dementia Rating Scale Sum of Boxes
CDR: Clinical Dementia Rating
CSDD: Cornell Scale for Depression in Dementia
CMMSE: Cantonese version of mini-mental state examination
CNKI: China National Knowledge Infrastructure
CNN: Cognitive control network
COPD: Chronic obstructive pulmonary disease
CVF: Category verbal fluency
CVFT: Category Verbal Fluency Test
DAD: Disability Assessment for Dementia
DS: Digit Span
DSC: Digit Symbol Coding
DSF: Digit Span forward
DSB: Digit Span backward
DST: Digit Span Test
DST-FB: Digit Span Test Forward–Backward
DSST: Digit Symbol Substitution Test
DTC: Dual-task cost
DQoL: Dementia Quality of Life
DR: Delayed recall
DLPFC: Dorsolateral prefrontal cortex
DMN: Default mode network
DSM-IV: Diagnostic and Statistical Manual of Mental Disorders
EQ-5D: EuroQoL 5-D Questionnaire
EQ-VAS: EuroQoL visual analogue scale
FaB: The Falls Behavioural scale
FAB: Frontal Assessment Battery
fALFF: Fractional amplitude of low-frequency fluctuations
FC: Functional connectivity
fNIRS: Functional near–infrared spectroscopy
fMRI: Functional magnetic resonance imaging
GAS-20: Geriatric Anxiety Scale with a maximum score of 20
GDS: Geriatric Depression Scale
GDS-15: Geriatric Depression Scale with a maximum score of 15
GRADE: The Grading of Recommendations, Assessment, Development and Evaluation
GUG: Get up and go
HK-MoCA: Montreal Cognitive Assessment Hong Kong version
HVLT: Hopkins Verbal Learning Test
HVLT-R: Hopkins Verbal Learning test–Revised
IL-10: Interleukin-10
IADL: Instrumental Activities of Daily Living
LM: Logical memory
Mattis DRS: Mattis Dementia Rating Scale
MC: Motor cortex
MCI: Mild cognitive impairment
MDRS: Mattis Dementia Rating Scale
MIC: Mini-Cog
MMSE: Mini-Mental State Examination
MoCA: Montreal Cognitive Assessment
MIC: Memory Inventory for Chinese Questionnaire
MIC: Memory Inventory for Chinese Questionnaire
MoCA: Montreal Cognitive Assessment
mPFC: Medial prefrontal cortex
MRI: Magnetic resonance imaging
MTG: Middle temporal gyrus
MOG: Middle occipital gyrus
MFG: Middle frontal gyrus
mPFC: Medial prefrontal cortex
NPI: Neuropsychiatric Inventory
NP: Neuropsychiatric
NRCT: Non-randomised controlled trial;
NRSI: Non-randomised studies of interventions
OC: Occipital cortex
PASE: Population Physical Activity Scale for the Elderly
PFC: Prefrontal cortex
PNRCT: Pilot non-randomised controlled trial
PPA: Physiological Profile Assessment
PD-39: Parkinson’s Disease Questionnaire-39
PASE: Physical activity scale for the elderly
PEF: Peak expiratory flow
PNCT: Prospective non
RAPA: Rapid Assessment of Physical Activity Scale
RCT: Randomised controlled trial
RMBT: Rivermead Behavioural Memory Test
rsFC: Resting state functional connectivity
rs-fMRI: Resting-state functional magnetic resonance imaging
RoB: The Cochrane risk of bias tool for randomised trials
sMRI: Structural magnetic resonance imaging
SF-36: RAND 36-Item Short-Form Health Survey-Medical Outcomes Study
SMC: Subjective Memory Complaints Scale
STS: Sit-to-stand
SR: Systematic reviews
TAT: Tinetti assessment tool
TMT: Trail Making Test
TMT: Trail Making Test
TSK: The Tampa Scale for Kinesiophobia
TCC: Tai Chi Chuan
TEA: Test of Everyday Attention
TMT: Trial-Making Test
TMT-A: Trial-Making Test-Part A
TMT-B: Trial-Making Test-Part B
TNF-α: Tumor necrosis factor-α
UG: Usual gait
VDS: Verbal descriptive scale
VS: Visual span
WAIS: Wechsler Adult Intelligence Scale
WOMAC: Western Ontario and McMaster Universities Osteoarthritis Index
