# Supplementary material for: Effects and mechanisms of Tai Chi on mild cognitive impairment and early-stage dementia: a scoping review
Source: Syst Rev. 2023 Oct 28;12:200. doi: 10.1186/s13643-023-02358-3 (PMC10612170; doi:10.1186/s13643-023-02358-3)
Supplement: Supplementary file 1 — Additional file 1: Tablet S1. Excluded studies that were screened for effectiveness analysis and reasons of exclusion. Table S2. Characteristics of included studies on the mechanisms of Tai Chi. [file 13643_2023_2358_MOESM1_ESM.docx]

**Supplementary Materials**

**Tablet S1.** Excluded studies that were screened for effectiveness analysis and reasons of exclusion

| **Study ID** | **Reason of exclusion** |
| --- | --- |
| Chan AW, Yu DS, Choi KC, Lee DT, Sit JW, Chan HY. Tai chi qigong as a means to improve night-time sleep quality among older adults with cognitive impairment: a pilot randomized controlled trial. Clin Interv Aging. 2016;11:1277-86. | Population: Suffering from sleep disturbance |
| Li F. Transforming traditional Tai Ji Quan techniques into integrative movement therapy-Tai Ji Quan: Moving for Better Balance. J Sport Health Sci. 2014;3(1):9-15. | Design: Non-RCT |
| Siu MY, Lee DTF. Effects of tai chi on cognition and instrumental activities of daily living in community dwelling older people with mild cognitive impairment. BMC Geriatr. 2018;18(1):37. | Design: Quasi-experimental study |
| Kasai JYT, Busse AL, Magaldi RM, Soci MA, Rosa PdM, Curiati JAE, et al. Effects of Tai Chi Chuan on cognition of elderly women with mild cognitive impairment. Einstein (Sao Paulo, Brazil). 2010;8(1):40-5. | Population: Healthy adults |
| Fogarty JN, Murphy KJ, McFarlane B, Montero-Odasso M, Wells J, Troyer AK, et al. Taoist Tai Chi and Memory Intervention for Individuals with Mild Cognitive Impairment. Journal of aging and physical activity. 2016;24(2):169-80. | Availability: No full text available |
| Guan XH, Li B. 管细红李博.太极拳训练对帕金森患者认知 功能及社会功能的影响.护士进修杂志 [Effects of tai chi training on cognitive and social functions of Parkinson’s patients]. J Nurses Train. 2016;31(18):1684–1686. Chinese. | Design: Quasi-experimental study Population: Irrelevant population |
| Wu TT, Wang YQ, Luo XR, et al. 汪亚群,罗相如,等.太极拳运 动对帕金森病患者认知功能及健康相关生活质量的影响.中 国康复 [Effects of Tai Chi exercise on cognition and healthrelated quality of life in patients with Parkinson’s disease]. Chin J Rehabil. 2018;33(2):95–97. | Design: Quasi-experimental study Population: Irrelevant population |
| Hsieh C-C, Lin P-S, Hsu W-C, Wang J-S, Huang Y-C, Lim A-Y, et al. The Effectiveness of a Virtual Reality-Based Tai Chi Exercise on Cognitive and Physical Function in Older Adults with Cognitive Impairment. Dementia and geriatric cognitive disorders. 2018;46(5-6):358-70. | Availability: No full text available |
| Cui L, Yin H, Lyu S, Shen Q, Wang Y, Li X, et al. Tai Chi Chuan vs General Aerobic Exercise in Brain Plasticity: A Multimodal MRI Study. Scientific reports. 2019;9(1):17264. | Design: Quasi-experimental study |
| Sun J, Kanagawa K, Sasaki J, Ooki S, Xu H, Wang L. Tai chi improves cognitive and physical function in the elderly: a randomized controlled trial. J Phys Ther Sci. 2015;27(5):1467-71. | Population: Healthy adults |
| Lavretsky H, Wetherell J, Smoski MJ, Varteresian T. Cultivation of well-being through mind-body interventions. American Journal of Geriatric Psychiatry. 2018;26(3 Supplement 1):S49-S50. | Population: Not related to cognitive impairment |

**Table S2.** Characteristics of included studies on the mechanisms of Tai Chi

| **Author, year** | **Imaging Protocol** | **Measurement** | **Study design** | **Sample size** | **Population and comparisons** |
| --- | --- | --- | --- | --- | --- |
| Tao J 2017a | Low frequency fluctuations | fALFF; fMRI | RCT | 61 | Older adults aged 50-70yrs randomised into Tai Chi and control groups. |
| Wei GX 2017 | Low frequency fluctuations | rs-fMRI; fALFF | Quasi experiment with matched control group | 40 | Tai Chi practitioners (aged 52.4+6.8yrs) versus demographically matched Tai Chi–naive healthy controls (aged 54.8+6.8yrs) |
| Xie H 2019 | Functional connectivity | fNIRS | Cross-sectional study | 55 | Experienced Chen–style Tai Chi practitioners (aged 65.01+2.61yrs) versus demographically matched Tai Chi–naive healthy controls (aged 65.34 +2.97yrs) |
| Tao J 2017b | Functional connectivity | fMRI | RCT | 61 | Older adults aged 50-70yrs randomised into Tai Chi and control groups. |
| Tao J 2016 | Functional connectivity | rs-fMRI | RCT | 62 | Healthy older volunteers (aged 50-70yrs) were randomized to the Tai Chi or control group. |
| Liu J 2019 | Functional connectivity | rs-fMRI | RCT | NI | Healthy, right-handed adults (aged 50-70yrs) who had not regularly participated in physical exercise for at least 1 year and had no history of psychiatric conditions were and randomized into a Tai Chi, Baduanjin or control group. |
| Cui L 2019 | Functional connectivity, volumetric change | sMRI & rs-fMRI | RCT | 36 | College students that were right-handed, with no history of psychiatric or neurological disease were randomly assigned to the Tai Chi (21.83+2.48yrs  ), aerobic exercise (21.92+2.28yrs), or control group (aged 21.75+2.45yrs). |
| Yue C 2020 | Volumetric change | rs-fMRI | Cross-sectional study | 42 | Health older women in Tai Chi (aged 62.90+2.38yrs) versus brisk walking exercise groups (63.27+3.58yrs) |
| Lv DY 2019 | Volumetric change | fMRI | Cross-sectional study (Master’s thesis) | 54 | Healthy long-term Tai Chi practitioners and healthy control without Tai Chi experience (aged 18-35yrs) |

***Abbreviations****: RCT, randomised control-trial; TCC, Tai Chi Chuan; AE, aerobic exercise; C, control; sMRI, structural magnetic resonance imaging; rs-fMRI, resting-state functional magnetic resonance imaging; MRI, magnetic resonance imaging; rsFC, resting state functional connectivity; fALFF, fractional amplitude of low-frequency fluctuations; fNIRS, functional near–infrared spectroscopy; fMRI, functional magnetic resonance imaging. NI, no information; yrs, years.*
